# Deep-learning based discrimination of pathologic complete response using MRI in HER2-positive and triple-negative breast cancer

**DOI:** 10.1038/s41598-024-74276-w

**Published:** 2024-10-04

**Authors:** Soo-Yeon Kim, Jinsu Lee, Nariya Cho, Young-Gon Kim

**Affiliations:** 1 Department of Radiology, Korea University Guro Hospital, Korea University College of Medicine, Seoul, Korea; 2 Innovative Medical Technology Research Institute, Seoul National University Hospital, Seoul, Republic of Korea; 3 Department of Radiology, Seoul National University Hospital, Seoul, Republic of Korea; 4 Department of Radiology, Seoul National College of Medicine, Seoul, Republic of Korea; 5 Institute of Radiation Medicine, Seoul National University Medical Research Center, Seoul, Republic of Korea; 6 Department of Transdisciplinary Medicine, Seoul National University Hospital, Seoul, Republic of Korea; 7 Department of Medicine, Seoul National University College of Medicine, 101 Daehak-ro, Jongno-gu, Seoul, 03080 Republic of Korea

**Keywords:** Breast cancer, Cancer imaging, Magnetic resonance imaging

## Abstract

Distinguishing between pathologic complete response and residual cancer after neoadjuvant chemotherapy (NAC) is crucial for treatment decisions, but the current imaging methods face challenges. To address this, we developed deep-learning models using post-NAC dynamic contrast-enhanced MRI and clinical data. A total of 852 women with human epidermal growth factor receptor 2 (HER2)-positive or triple-negative breast cancer were randomly divided into a training set (*n* = 724) and a validation set (*n* = 128). A 3D convolutional neural network model was trained on the training set and validated independently. The main models were developed using cropped MRI images, but models using uncropped whole images were also explored. The delayed-phase model demonstrated superior performance compared to the early-phase model (area under the receiver operating characteristic curve [AUC] = 0.74 vs. 0.69, *P* = 0.013) and the combined model integrating multiple dynamic phases and clinical data (AUC = 0.74 vs. 0.70, *P* = 0.022). Deep-learning models using uncropped whole images exhibited inferior performance, with AUCs ranging from 0.45 to 0.54. Further refinement and external validation are necessary for enhanced accuracy.

**Supplementary Information:**

The online version contains supplementary material available at 10.1038/s41598-024-74276-w.

## Introduction

Neoadjuvant chemotherapy (NAC) for breast cancer reduces primary tumor size and axillary lymph node metastatic burden, making breast-conserving surgery and sentinel lymph node biopsy viable alternatives to mastectomy and axillary lymph node dissection^1,2^. Exceptional responders can achieve a pathologic complete response (pCR), which is a surrogate marker indicative of an excellent prognosis in triple-negative and human epidermal growth factor receptor type 2 (HER2)-positive breast cancer^3^.

After the completion of NAC, evaluation of residual tumors using imaging examinations is crucial to determine the method and the extent of surgical intervention. Among imaging examinations, dynamic contrast-enhanced (DCE) MRI is the most accurate, although it is not perfect^4^. Overestimation or underestimation can occur because of decreased vascularity, fragmentation of tumors, fibrosis, and inflammation caused by chemotherapy^4^. In a systemic review by Lobbes et al., the median correlation coefficient was 0.70 (range, 0.21–0.98) between tumor size measured on MRI and tumor size reported on surgical pathology^5^. In meta-analyses, the pooled sensitivity and specificity of post-NAC MRI showed a wide range of heterogeneous values, suggesting difficulties in assessing residual tumors and the potential for inter-observer variability^6–10^.

For patients who achieve pCR, the added value of breast surgery remains uncertain. Therefore, several clinical trials are currently underway to evaluate the feasibility and safety of obviating the need for surgery in patients who achieve radiologic CR^11–13^. As imaging methods are not sufficiently precise, these trials use vacuum-assisted biopsy to predict pCR^11–13^. However, biopsy has limitations such as false-negative diagnosis, the need for skilled radiologists, and rare complications such as bleeding and pneumothorax. Therefore, the development of a non-invasive imaging method to accurately predict pCR is a timely and clinically relevant subject.

Deep-learning techniques based on MRI have been used to predict pCR more accurately^14,15^. Most studies have predicted pCR using pretreatment or early treatment MRI^16–22^. Until now, few studies have classified pCR versus non-pCR using MRI after NAC completion^23,24^. Additionally, most studies have pooled all phases of DCE-MRI instead of evaluating the performance of each phase independently^16–24^. Using the best-performing single dynamic phase as input may not only improve the model performance but also reduce the computational burden compared to using all dynamic phases as inputs. Furthermore, most studies have pooled all breast cancer subtypes^16,18–21,23,24^. Breast cancers have heterogeneous properties and different pCR rates depending on their subtypes^3^. Luminal (hormone receptor-positive and HER2-negative) breast cancers usually undergo NAC in the locally advanced stage, with pCR rates being low, approximately 5–15%. NAC is performed for these patients to reduce tumor extent rather than to achieve pCR. In contrast, HER2-positive and triple-negative breast cancers undergo NAC not only in the locally advanced stage but also in earlier stages, with pCR rates being significantly higher, up to 40–60%. Consequently, clinical trials aimed at omitting surgery after NAC often include HER2-positive and triple-negative breast cancers and exclude luminal breast cancers^13^. Accordingly, subtype-specific prediction models have greater clinical impact.

Therefore, we developed a deep-learning model for discriminating pCR status using DCE-MRI performed after completion of NAC in patients with HER2-positive and triple-negative breast cancers.

## Materials and methods

This retrospective single-center study was approved by the Institutional Review Board of Seoul National University Hospital (No. 2303-028-1409). The requirement for informed consent was waived by the Institutional Review Board, because of the retrospective study design. All methods were conducted in accordance with the Declaration of Helsinki.

### Study patients

**Fig. 1:**
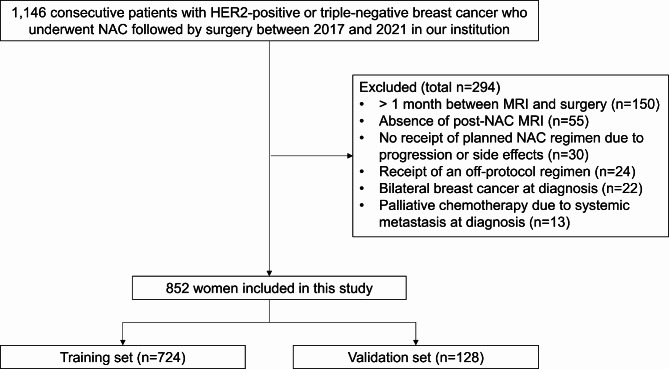
Flow chart of the study. HER2 = human epidermal growth factor receptor 2, NAC = neoadjuvant chemotherapy.

The inclusion criteria comprised consecutive patients diagnosed with HER2-positive or triple-negative breast cancer who underwent NAC followed by surgery from 2017 to 2021 at our institution. Exclusion criteria included patients who did not receive the planned NAC regimen due to disease progression or adverse effects, those who received an off-protocol regimen, those lacking post-NAC MRI, those with a time interval of more than one month between post-NAC MRI and surgery, those diagnosed with bilateral breast cancer, and those who underwent palliative chemotherapy due to systemic metastasis at diagnosis. From our institution’s breast cancer registry, we identified 1,146 patients with HER2-positive or triple-negative breast cancer who underwent NAC followed by surgery between 2017 and 2021. Among them, 294 women were excluded because of the following reasons: more than a one-month interval between post-NAC MRI and surgery (*n* = 150), absence of post-NAC MRI (*n* = 55), no receipt of planned NAC regimen due to progression or side effects (*n* = 30), receipt of an off-protocol regimen (*n* = 24), bilateral breast cancer at diagnosis (*n* = 22), and palliative chemotherapy due to systemic metastasis at diagnosis (*n* = 13). Consequently, a total of 852 women (mean age, 51 ± 10 years) were included in this study (Fig. 1). Among the 852 women included in the study, 526 had HER2-positive breast cancers, and 326 had triple-negative breast cancers.

## NAC regimen

During the study period, patients with HER2-positive breast cancers were administered one of two chemotherapy regimens at the discretion of the oncology specialists with 5–25 years of experiences: AC#4-DH#4 (four cycles of doxorubicin [60 mg/m^2^] plus cyclophosphamide [600 mg/m^2^] followed by four cycles of docetaxel [75 mg/m^2^] plus herceptin [8 mg/kg loading dose followed by 6 mg/kg]) or TCHP#6 (six cycles of docetaxel [75 mg/m^2^] plus carboplatin [area under the plasma concentration-time curve 5] plus herceptin [8 mg/kg loading dose followed by 6 mg/kg] plus pertuzumab [840 mg loading dose followed by 420 mg]). For patients with triple-negative breast cancer, the choice between the following two chemotherapy regimens was made: AC#4-D#4 (four cycles of doxorubicin [60 mg/m^2^] plus cyclophosphamide [600 mg/m^2^] followed by four cycles of docetaxel [75 mg/m^2^]) or AD#6 (six cycles of doxorubicin [50 mg/m^2^] plus docetaxel [75 mg/m^2^]). Specifically, a total of 526 women with HER2-positive breast cancers received either AC#4-DH#4 (*n* = 129) or TCHP#6 (*n* = 397), while a total of 326 women with triple-negative breast cancers received either AC#4-D#4 (*n* = 271) or AD#6 (*n* = 55).

## Clinical data collection

The following clinical data were collected from the electronic medical records: age (years), tumor size at baseline MRI (cm), clinical T stage (1–4), clinical N stage (0–3), estrogen receptor (ER) status (negative or positive), progesterone receptor (PR) status (negative or positive), HER2 status (negative or positive), Ki-67 index (%), histologic grade (low or intermediate vs. high), and histologic type (ductal vs. others). Data on ER, PR, HER2, Ki-67, histologic grade, and histologic type were obtained from the biopsy specimens before starting NAC. ER and PR positivity were defined as nuclear staining in 1% or more cancer cells by immunohistochemistry. HER2-positivity was defined as either a HER2 score of 3 + by immunohistochemistry or the presence of gene amplification by fluorescence in situ hybridization in tumors with a HER2 score of 2+.

### Reference standard

pCR was defined as the complete absence of invasive and in situ tumors in the breast based on postoperative pathological examination, irrespective of the axillary nodal status (i.e., ypT0), for use in clinical trials to obviate the need for breast surgery.

## DCE-MRI protocol and region-of-interest annotation

Breast MRI was performed using a 3T MRI scanner, either Philips Ingenia (55% [471 of 852]) or Siemens Skyra (45% [381 of 852]). The routine MRI protocol sequentially consisted of an axial fat-suppressed T2-weighted sequence, DCE-MRI with an axial 3D fat-suppressed T1-weighted gradient echo sequence, sagittal fat-suppressed T1-weighted sequence, axial fat-suppressed T1-weighted sequence for the axillary regions, and axial diffusion-weighted sequence. DCE-MRI was performed using one pre-and five post-contrast dynamic series (Table E1 for the detailed parameters). For DCE-MRI, gadobutrol (Gadovist, Bayer Healthcare, Berlin, Germany) was administered at a dose of 0.1 mmol/kg and at a rate of 2 mL/sec, followed by a 20 mL saline flush. Post-contrast dynamic images were acquired 90, 180, 270, 360, and 450 s after contrast injection. The subtraction images were created by subtracting pre-enhanced images from contrast-enhanced images. In our institution, we routinely acquire subtraction images from the first, third, and fifth dynamic phases, while we do not obtain subtraction images from the second and fourth dynamic phases. In this study, the subtraction images from the first, third, and fifth dynamic phases (hereafter referred to as sub1, sub3, and sub5, respectively) were used to create a deep-learning model.

To create a cropped deep-learning model for the region of interest (ROI) of a residual-enhancing lesion, a dedicated breast radiologist with 11 years of experience annotated the ROIs on the fifth dynamic subtraction images. Three rectangular shaped ROIs were placed at the three points (beginning, middle, and ending) of a residual enhancing lesion, fitting to the lesion. For absence of residual enhancement, ROIs were placed according to the location and size of the initial tumor on pre-NAC MRI and the location of the inserted clip. To specify, the rectangular-shaped ROIs were positioned at three key points along the axial dimension of the lesion: the starting point, the middle point (representing the largest rectangular), and the ending point. These ROIs could encompass not only the target lesion but also a small area of surrounding tissue due to its shape. Engineers then utilized these three boxes to generate a 3D voxel ROI, which served as input for the deep learning models. The choice of using the fifth dynamic phase (the last phase of DCE-MRI) was based on the tendency for the extent of the residual tumor to be more accurately and prominently visualized on delayed phases compared to early phases, due to the effect of NAC on delaying enhancement^25,26^. The 3D voxel ROI obtained from the fifth dynamic phase was subsequently applied to the first and third subtraction images.

## Preprocessing

MR image preprocessing was performed using the SimpleITK Python package (V2.1) as follows: First, the voxel spacing values were equalized using linear interpolation in 3D. Second, the MR signal intensity was normalized using multidimensional contrast-limited adaptive histogram equalization to mitigate intensity diversity and standardize image intensities^17^. Third, to make the input sizes equal, the MR images were cropped to the same size of 72 × 66 × 22 voxels, which was determined to be the median size of all 3D voxel ROIs. Choosing the median size rather than the largest size was to maintain the higher resolution.

### Data splitting

A total of 852 patients were divided into a training set (*n* = 724) and validation set (*n* = 128) at a ratio of 8.5:1.5. The division between the two sets was determined using a stratified split based on pCR versus non-pCR status, which is the prediction target of our study. Other clinical information was not considered during the division process and thus was randomly distributed. The deep-learning model was trained using 5-fold cross validation in the training set, where four-fifths of the training set was used for training the model and the remaining one-fifth was used to tune the hyperparameters of the model. The performance of the developed deep-learning model was validated in the validation set.

## Development of clinical model

Based on the aforementioned clinical data (age, initial tumor size, clinical stage, ER, PR, HER2, Ki-67, histologic grade, and type), five clinical models were developed using a simple deep-learning method called multilayer perception (MLP) and four machine learning methods: Logistic Regression (LR), Random Forest (RF), Support Vector Machine (SVM), and XGBoost (XGB). The detailed parameters of each clinical model were optimized using the Optuna Python package (V3.0). For the MLP model, five parameters were considered for optimization, including the number of layers, the type of activation function, and the solver for weight optimization. For the LR model, the parameters considered were penalty (L1 or L2), C as a regularization parameter, and the maximum number of iterations. For the RF model, eight parameters were considered, including the function to measure the quality of a split, the number of trees, and the maximum depth of the tree. For the SVM model, six parameters were considered, including C as a regularization parameter, the kernel type, and the degree of the kernel function. For the XGB model, ten parameters were considered, including the booster type, the maximum depth of a tree, and lambda or alpha as regularization parameters.

## Deep-learning model architecture

**Fig. 2:**
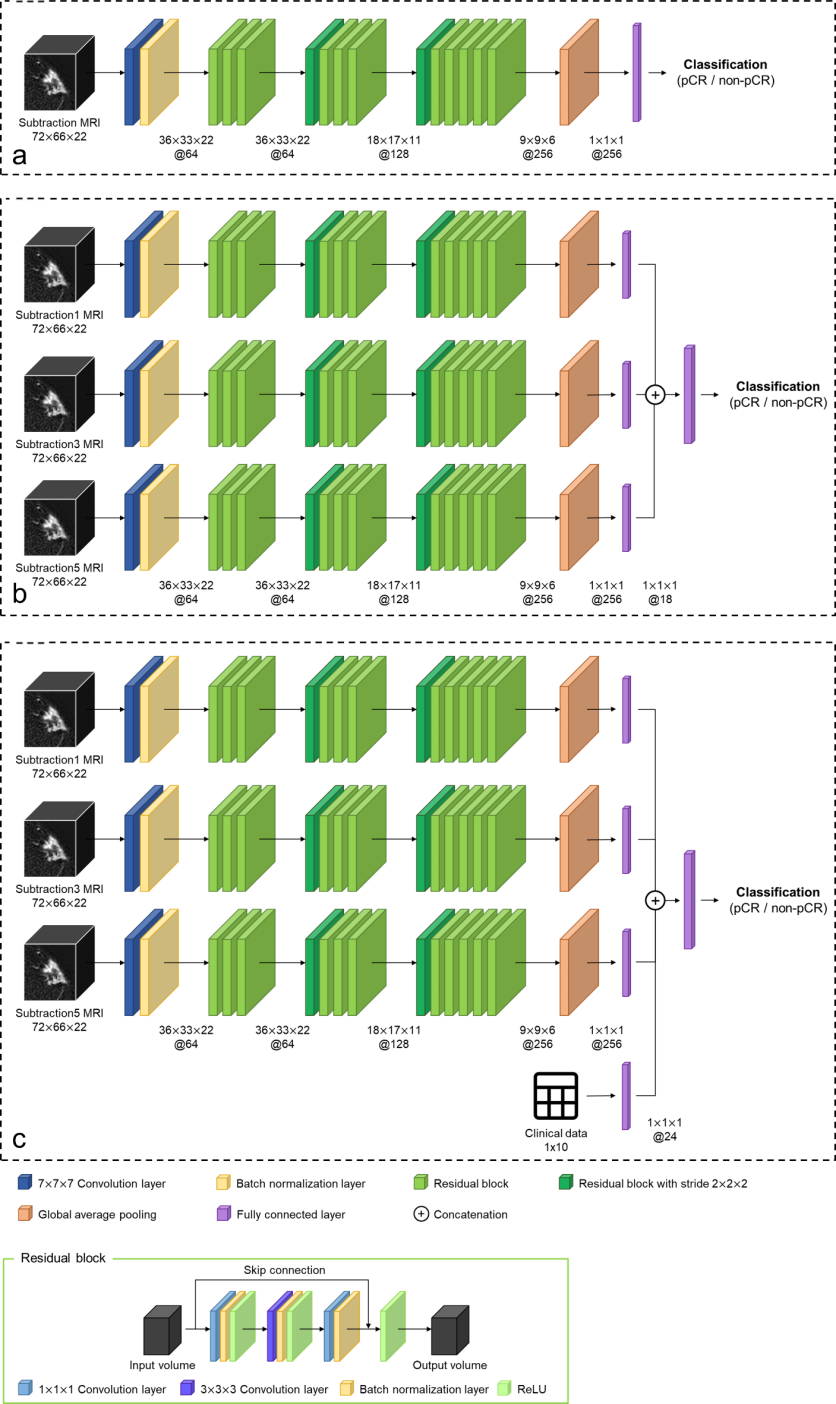
Deep-learning model architecture. (a) single-phase MRI (sub1, sub3, or sub5), (b) multiple-phase MRI (sub1 + sub3 + sub5), (c) combined MRI and clinical data (sub + sub3 + sub5 + clinical data). sub1 = subtraction images of the first dynamic phase. sub3 = subtraction images of the third dynamic phase. sub5 = subtraction images of the fifth dynamic phase.

Deep-learning models were created based on the 3D convolutional neural network (CNN) ResNet50 model architecture by Hara et al., with modifications considering data size^27^. Firstly, the max pooling layer with size of 3 × 3 × 3 in the first layer block was removed in order to retain the information of input volume. Additionally, the fifth layer block with three 512-dimensional convolution layers was excluded in our modified model, as it was deemed that the previous four layer blocks were sufficient for extracting the features of the input 3D image. Furthermore, the convolutional stride with a size of 2 × 2 × 2 was applied only at the first convolution layer of third and fourth layer block, which prevented the extracted 3D feature map from becoming too small in size. Three types of deep learning models were created using different input data as follows: (a) a single dynamic phase of DCE-MRI (sub1, sub3, or sub5), (b) multiple dynamic phases of DCE-MRI (sub1 + sub3 + sub5), and (c) combined MRI and clinical data (sub1 + sub3 + sub5 + clinical data) (Fig. 2).

Cropped and uncropped models were created. The cropped model was created using the ROI annotated by the radiologist, as mentioned above. An uncropped model was created using all the MR images without ROI annotation.

The single-phase model used the subtraction images of the single dynamic phases of DCE-MRI as the input (sub1, sub3, or sub5). The single-phase model comprised three convolutional stages, following an initial 7 × 7 × 7 convolutional layer and a batch normalization layer. The first convolutional stage encompassed three residual blocks with 64-dimension. The second stage involved four residual blocks with 128-dimension, with the initial block featuring a 2 × 2 × 2 stride. Similarly, the third stage comprised six residual blocks with 256-dimension, with an initial block of 2 × 2 × 2 strides. Finally, a global average pooling layer was applied to extract the features as a 256-dimensional vector. The architecture of this model is shown in Fig. 1a.

The multiple-phase model used all three phases of the MRI as the input. MRI images from each phase were entered into separate single models, and the resulting 6-dimensional features were concatenated to create a single 18-dimensional feature. This concatenated feature was then fed into a fully connected dense layer, similar to the single model. The architecture of the model is shown in Fig. 1b.

The combined model utilized not only the three phases of MRI but also clinical data as inputs. Similar to the multiple-phase MRI model, 6-dimensional features were extracted from each phase of the MRI. In addition, the clinical data were extracted as 6-dimensional feature using fully connected layers. The subsequent processes, including feature concatenation, were the same as those used for the multiple-phase MRI model. The architecture of the model is shown in Fig. 1c. To ensure objectivity in determining the optimal hyperparameters, we employed an automated grid search methodology utilizing the hyperparameter optimization framework Optuna. Through this approach, we found that reducing the features to 6 dimensions yielded the best performance for our study. We did not perform an independent test before building a combined model, as we thought that the grid search-based parameter optimization process used in this study would minimize the correlation between the features. The deep-learning models were implemented using Python 3.6, TensorFlow 2.9, and Keras 2.9. The training was performed using an NVIDIA RTX 3090, with a learning rate of 2e-7, batch size of 16, and early stopping. The learning rate and batch size were set to 2e-7 and 16, respectively, as these values empirically demonstrated the best performance. Early stopping was applied with a patience of 30 steps, meaning that training was stopped if the validation loss did not improve for 30 consecutive steps. Data augmentation was performed by flipping the image in the sagittal plane to improve model generalization. This augmentation effectively increases the diversity of the training data by simulating different orientations of the breast MRIs. This method helps the model become more robust to variations in patient positioning and anatomical differences.

### Visualization of the results

To provide interpretability of the model, we used gradient class activation maps (Grad-CAMs). Grad-CAM is a technique used in the fields of computer vision and deep-learning to understand the parts of an input image that are most influential in a neural network’s decision-making process for a particular class or category^28^.

### Statistical analysis

Clinical characteristics of training set and validation set were compared using the Mann-Whitney U test for continuous variables and the χ2 test or Fisher’s exact test for categorical variables. The area under the receiver operating characteristic curve (AUC), accuracy, sensitivity, specificity, positive predictive value, and negative predictive value of each model were calculated for the validation set at the patient level. These metrics were derived from the model’s predictions compared to the ground truth labels. Among the five clinical models, the model with the maximum AUC was selected as the representative clinical model. AUC of each model was compared using DeLong method^29^.

## Results

### Patient characteristics

Table 1 shows the clinical and pathological characteristics of all patients (*n* = 852), patients in the training set (*n* = 724), and patients in the validation set (*n* = 128). Most characteristics were similar between the training and validation sets, although tumor size on pre-NAC MRI and clinical N stage were significantly different between the two sets (*P* = 0.014 and *P* = 0.003, respectively). The pCR rate was 35.9% (260 of 724) in the training set and 39.1% (50 of 128) in the validation set.

**Table 1:** Clinical and pathological characteristics of training set and validation set.

Characteristics	Total (*n* = 852)	Training set (*n* = 724)	Validation set (*n* = 128)	*P* value
Age (years)	51 ± 10	51 ± 10	52 ± 10	0.483
Tumor size on pre-NAC MRI (cm)	4.1 ± 2.1	4.2 ± 2.1	3.7 ± 1.8	0.014
Clinical T stage				0.194
T1	48 (5.6)	39 (5.4)	9 (7.0)	
T2	575 (67.5)	481 (66.4)	94 (73.4)	
T3	141 (16.5)	124 (17.1)	17 (13.3)	
T4	88 (10.3)	80 (11.0)	8 (6.3)	
Clinical N stage				0.003
N0	124 (14.6)	94 (13.0)	30 (23.4)	
N1	358 (42.0)	303 (41.9)	55 (43.0)	
N2	240 (28.2)	217 (30.0)	23 (18.0)	
N3	130 (15.3)	110 (15.2)	20 (15.6)	
Estrogen receptor status Negative Positive	583 (68.4) 269 (31.6)	494 (68.2) 230 (31.8)	89 (69.5) 39 (30.5)	0.771
Progesterone receptor status Negative Positive	693 (81.3) 159 (18.7)	586 (80.9) 138 (19.1)	107 (83.6) 21 (16.4)	0.477
HER2 status Negative Positive	326 (38.3) 526 (61.7)	271 (37.4) 453 (62.6)	55 (43.0) 73 (57.0)	0.235
Ki-67 index (%)	22 ± 20	22 ± 20	22 ± 22	0.919
Histologic grade				0.091
Low or intermediate	381 (44.7)	315 (43.5)	66 (51.6)	
High	471 (55.3)	409 (56.5)	62 (48.4)	
Histologic type				0.475
Ductal	831 (97.5)	705 (97.4)	126 (98.4)	
Others	21 (2.5)	19 (2.6)	2 (1.6)	
Pathologic complete response				0.495
No	542 (63.6)	464 (64.1)	78 (60.9)	
Yes	310 (36.4)	260 (35.9)	50 (39.1)	

Note. – For continuous variables, data are means with standard deviations (SD) in parentheses. For categorical variables, data are number of women with percentages in parentheses. HER2 = human epidermal growth factor receptor 2.

### Performance of the clinical model

Five clinical models were developed using one deep-learning technique and four machine learning techniques, and the performance of each model was compared (Table E2). The AUC of the five clinical models were low, ranging from 0.59 to 0.63. Among the five clinical models, the model developed using the XGBoost technique showed the highest AUC value of 0.63 (95% CI: 0.59–0.67), thus was selected as the representative clinical model.

### Performance of the deep-learning imaging models using the cropped images

**Fig. 3:**
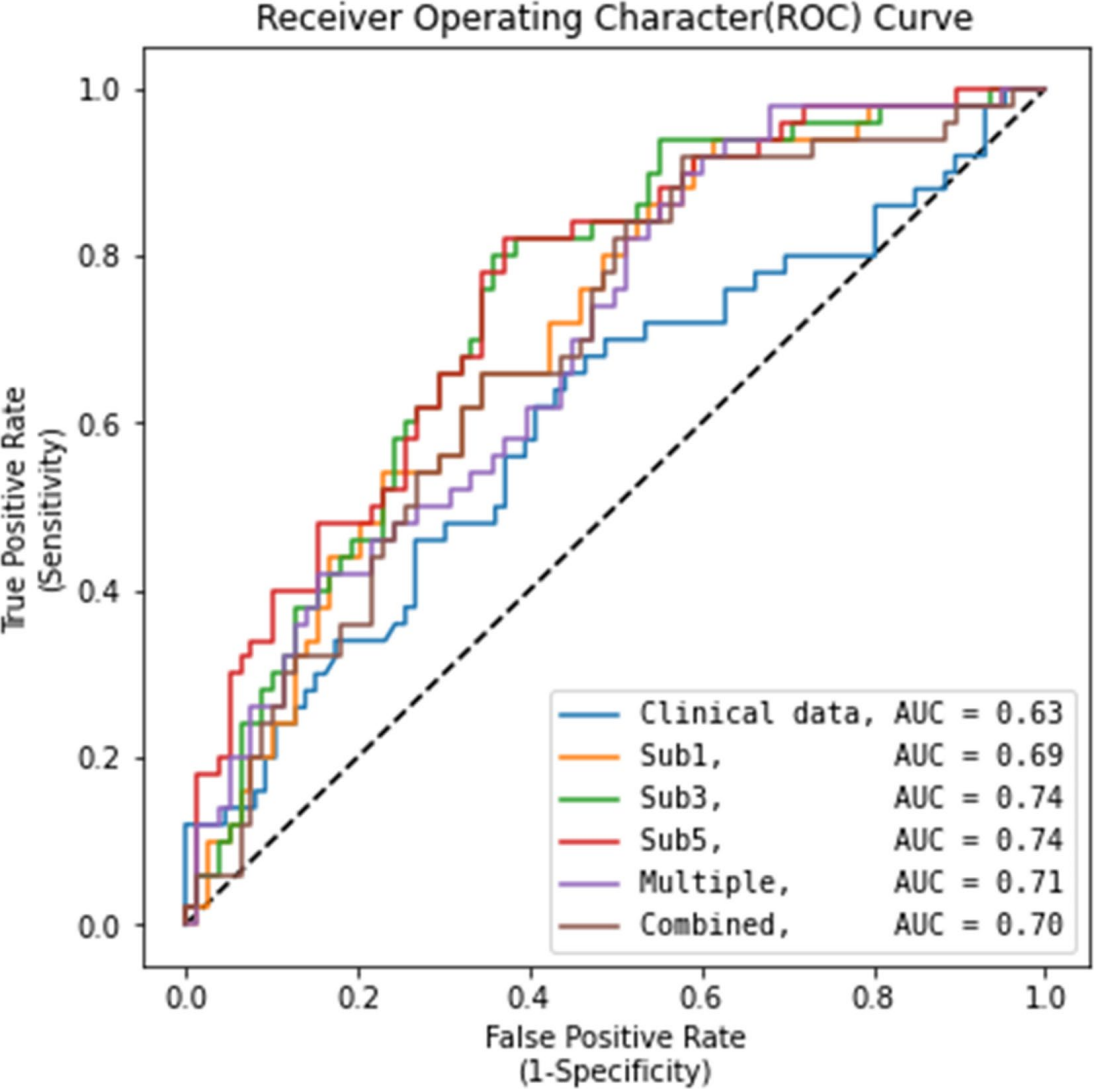
Receiver operating characteristic curves showing the AUC values of different deep-learning models in the validation set. Sub1 = subtraction images of the first dynamic phase of dynamic contrast-enhanced (DCE)-MRI. Sub3 = subtraction images of the third dynamic phase of DCE-MRI. Sub5 = subtraction images of the fifth dynamic phase of DCE-MRI. Multiple = Sub 1 + Sub 3 + Sub 5. Combined = Sub 1 + Sub 3 + Sub 5 + Clinical data.

**Fig. 4:**
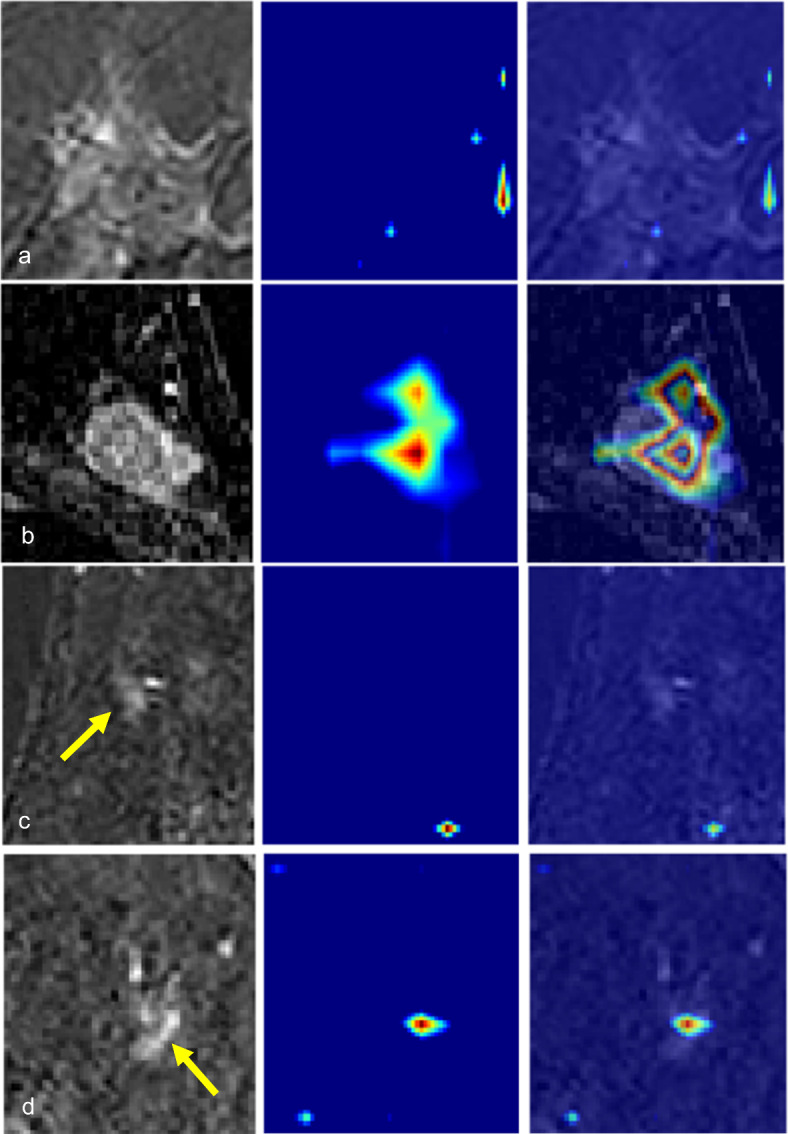
Visualization of the activation of the convolutional layer using GradCAM. The first column shows center slices of residual enhancing lesions or the tumor bed in the subtraction images of the 3rd dynamic phase of dynamic contrast-enhanced MRI. These are preprocessed cropped images. The second column shows the corresponding GradCAM activation heat map. The third column presents the overlaid image of the previous two columns. Representative images were selected based on the GradCAM results, with pathologic complete response (pCR) status as the reference standard, categorized as true-positive, true-negative, false-positive and false-negative. True positive: A 55-year-old woman with human epidermal growth factor type 2 (HER2)-positive breast cancer achieved pCR. No enhancement was observed in the tumor bed. GradCAM was not activated in the tumor bed. True negative: A 40-year-old woman with triple-negative breast cancer did not achieve pCR. There remained a 2 cm enhancing mass, and GradCAM was activated for the enhancing area. False positive: A 31-year-old woman with triple-negative breast cancer did not achieve pCR. A 0.9 cm enhancing mass (arrow) remained, and GradCAM was not activated. False negative: A 42-year-old woman with HER2-positive breast cancer achieved pCR. There remained a 0.8 cm subtle enhancement (arrow), and GradCAM was activated for the enhancing lesion.

The performances of the deep-learning imaging models using cropped images (with segmentations) in the validation set are provided in Table 2; Fig. 3. The AUC values of deep-learning models using the single-phase of DCE-MRI were 0.69 (95% CI: 0.68–0.70) for sub1, 0.74 (95% CI: 0.74–0.74) for sub3, and 0.74 (95% CI: 0.73–0.75) for sub5. The sub3 and sub5 model exhibited identical AUC values up to the second decimal place (0.74) but differed at the third decimal place (0.743 and 0.741, respectively). The sub3 model showed significantly higher AUC value than the sub1 model (0.74 vs. 0.69, *P* = 0.013) and the clinical model using the XGBoost technique (0.74 vs. 0.63, *P* < 0.001). The AUC value of the multiple-phase model was 0.71 (95% CI: 0.70–0.72), comparable with that of the sub3 model (0.71 vs. 0.74, *P* = 0.087). The AUC value of the combined model was 0.70 (95% CI: 0.69–0.71), significantly lower than that of the sub3 model (0.70 vs. 0.74, *P* = 0.022). The sensitivity of the deep-learning models using cropped images ranged from 0.44 to 0.56. The specificity ranged from 0.75 to 0.79. Grad-CAM applied to the sub3 model is shown in Fig. 4 and Figure E1, providing interpretability of the results. From the Grad-CAM results, we confirmed that the 3D CNN model predicted pCR or non-pCR status based on MRI information of the tumor bed. For example, in the true negative case, the activation of the CNN model occurred in the region corresponding to the residual enhancing lesion in the MRI image, as seen in Fig. 4b and Figure E1. On the other hand, in the case of true positives, the CNN model was not activated in the tumor bed region but was activated at the image border or activated randomly throughout the image, as seen in Fig. 4a.

**Table 2:** Performance of the deep-learning imaging models using cropped images in the validation set.

Input	AUC	*P* value	Accuracy	Sensitivity	Specificity	PPV	NPV
Sub1	0.69 (0.68–0.70)	0.013	0.63 (0.61–0.65)	0.44 (0.39–0.49)	0.76 (0.74–0.78)	0.54 (0.51–0.57)	0.68 (0.67–0.69)
Sub3	0.74 (0.74–0.74)	Reference	0.7 (0.69–0.71)	0.56 (0.53–0.59)	0.79 (0.77–0.81)	0.63 (0.62–0.64)	0.74 (0.73–0.75)
Sub5	0.74 (0.73–0.75)	0.873	0.67 (0.66–0.68)	0.52 (0.49–0.55)	0.76 (0.73–0.79)	0.58 (0.56–0.60)	0.71 (0.70–0.72)
Multiple phase	0.71 (0.70–0.72)	0.087	0.66 (0.65–0.67)	0.48 (0.40–0.56)	0.78 (0.72–0.84)	0.64 (0.57–0.71)	0.71 (0.69–0.73)
Combined	0.70 (0.69–0.71)	0.022	0.65 (0.64–0.66)	0.51 (0.46–0.56)	0.75 (0.71–0.79)	0.58 (0.55–0.61)	0.71 (0.70–0.72)

Note. – 95% confidence intervals in parentheses. AUC = area under the receiver operating characteristic curve, NPV = negative predictive value, PPV = positive predictive value.

Sub1 = subtraction images of the first dynamic phase of dynamic contrast-enhanced (DCE)-MRI.

Sub3 = subtraction images of the third dynamic phase of DCE-MRI.

Sub5 = subtraction images of the fifth dynamic phase of DCE-MRI.

Multiple phase = Sub 1 + Sub 3 + Sub 5.

Combined = Sub 1 + Sub 3 + Sub 5 + Clinical data.

### Performance of the deep-learning imaging models using whole images

The performances of the deep-learning models using whole images (without segmentations) in the validation set are listed in Table 3. Overall, AUC values were low, ranging from 0.45 to 0.54. The sub3 model showed the highest AUC value of 0.54 (95% CI: 0.51–0.57) among the models listed in the Table 3. The multiple-phase model and the combined model showed AUC less than 0.5 (0.47 and 0.45, respectively). The sensitivity ranged from 0 to 0.03. The specificity ranged from 0.87 to 1.00.

**Table 3:** Performance of the deep-learning imaging models using whole images in the validation set.

Input	AUC	Accuracy	Sensitivity	Specificity	PPV	NPV
Sub1	0.51 (0.46–0.56)	0.61 (0.60–0.62)	0.03 (-0.03-0.09)	0.99 (0.97-1.00)	0.13 (-0.12-0.38)	0.61 (0.60–0.62)
Sub3	0.54 (0.51–0.57)	0.61 (0.61–0.61)	0.01 (-0.01-0.03)	0.99 (0.98-1.00)	0.13 (-0.14-0.40)	0.61 (0.61–0.61)
Sub5	0.52 (0.47–0.57)	0.61 (0.61–0.61)	0 (0.00–0.00)	1 (1.00–1.00)	0 (0.00–0.00)	0.61 (0.61–0.61)
Multiple phase	0.47 (0.40–0.54)	0.57 (0.52–0.62)	0.11 (0.02–0.20)	0.87 (0.74-1.00)	0.22 (0.03–0.41)	0.6 (0.58–0.62)
Combined	0.45 (0.40–0.50)	0.6 (0.60–0.61)	0 (0.00-0.01)	0.99 (0.97-1.00)	0.04 (-0.04-0.12)	0.61 (0.60–0.61)

Note. – 95% confidence intervals in parentheses. AUC = area under the receiver operating characteristic curve, NPV = negative predictive value, PPV = positive predictive value.

Sub1 = subtraction images of the first dynamic phase of dynamic contrast-enhanced (DCE)-MRI.

Sub3 = subtraction images of the third dynamic phase of DCE-MRI.

Sub5 = subtraction images of the fifth dynamic phase of DCE-MRI.

Multiple phase = Sub 1 + Sub 3 + Sub 5.

Combined = Sub 1 + Sub 3 + Sub 5 + Clinical data.

## Discussion

The precise discrimination of pCR through MRI acquired after the completion of NAC is becoming increasingly important in patients with HER2-positivie and triple-negative breast cancers. This importance is underscored by the high pCR rates in these subtypes and the increasing interest in clinical trials that omit surgery in exceptional responders. We developed a deep-learning model based on DCE-MRI and clinical information in the training set (*n* = 724) and evaluated its performance in the validation set (*n* = 124). We found that the deep-learning model based on the delayed-phase, rather than the early-phase of DCE-MRI, showed better performance in discriminating pCR. Moreover, the single delayed-phase model showed comparable performance to the multiple-phase model and better performance than the combined model using multiple phases of DCE-MRI and clinical information. Additionally, deep learning models that input whole MR images without segmentation exhibited results that were scarcely trained.

To our knowledge, this was the first study to develop separate deep-learning MRI models for early and delayed phases in discriminating pCR from residual cancer. To date, only two studies have developed deep-learning models for pCR prediction using post-NAC DCE-MRI^23,24^. These studies collectively utilized all phases of DCE-MRI without distinguishing between early- and delayed- phases^23,24^. The comparison of the study design between the two prior studies and our study is presented in Table E3 of the supplemental material. We observed that the delayed-phase model outperformed the early-phase model, which is in line with findings from prior studies^25,26^. Santamaria et al. found that the absence of enhancement on delayed-phase MRI increased the probability of pCR by 28 times compared to the presence of enhancement^25^. Kim et al. demonstrated a stronger correlation between tumor size measured in the delayed-phase than in the early-phase of DCE-MRI and the actual tumor size found in surgical specimens^26^. Moreover, our study found comparable performance between the single delayed-phase model and the multi-phase model. This comparable performance may be due to the limited data size, which possibly hindered the full optimization of the multi-phase model’s parameters. With a larger dataset, the model might better utilize the features from multiple phases. Nontheless, our findings suggest that a deep-learning model using single delayed-phase MR images may suffice, obviating the need for multiple DCE-MRI phases. This approach could mitigate the computational demands and minimize potential registration errors.

Despite being developed using the versatile clinical information listed in Table 1, our clinical models generally showed poor discrimination performance, with AUC values ranging from 0.59 to 0.63. Furthermore, the combined model, which integrated multiple phases of DCE-MRI with clinical information, yielded lower performance than the single delayed-phase model. These results highlight the limited performance of clinical information in discriminating pCR from residual cancer after NAC. However, further studies with larger data sizes are needed, as these findings seem inconsistent with previous studies demonstrating the superiority of the combined model utilizing both MRI and clinical information over the MRI-only model^19,24^. The disparities in breast cancer subtypes across various studies likely contribute to this inconsistency. Previous studies encompassed all breast cancer subtypes, whereas our study focused solely on the HER2-positive and triple-negative subtypes, excluding the luminal (hormone receptor-positive and HER2-negative) subtypes^19,24^. Breast cancer subtypes are expected to possess substantial predictive power for pCR within the realm of clinical information. This predictive capability is likely attributed to the marked differences in pCR rates between the luminal and non-luminal subtypes. However, given that our study solely included non-luminal subtypes while excluding luminal subtypes, it was presumed that the predictive capacity of subtypes within our study cohort had diminished.

In our study, the deep-learning models using unsegmented whole MR images exhibited AUC values ranging from 0.45 to 0.54, indicating limited efficacy. The decreased performance observed when using whole images without segmentation may be attributed to the model learning unnecessary and excessive information that is not relevant for accurate judgment. Including unnecessary regions can introduce noise and irrelevant information, potentially confusing the model and degrading its performance. Models trained on such data may exhibit reduced accuracy and robustness^30^. These findings are inconsistent with those of Dammu et al.^24^ Their study demonstrated an acceptable AUC of 0.78 for a deep-learning model using unsegmented whole MR images in differentiating pCR. Compared with the utilization of images segmented by human observers, the use of unsegmented whole images has the potential to alleviate issues related to labor intensity and inter-observer variability. However, as our study demonstrates, this might not lead to effective learning and could also lead to an increase in computational burden and cost. Given the insufficient number of studies addressing this issue, further research is required.

Furthermore, the MRI models exhibited relatively high specificity but low sensitivity, aligning with earlier research findings^19,24^. In simpler terms, there were instances where MRI incorrectly classified pCR as non-pCR. This could be attributed to the presence of slight enhancements caused by factors like inflammation, granulation, or fibrosis, even in cases of pCR status^31^.

Our study has several limitations. First, it was a single-center study. External validation is required to confirm the generalizability of our model. Second, we did not use multiparametric MRI to develop the deep-learning model. We used subtraction images from DCE-MRI and did not use other MRI sequences such as T2-weighted imaging or diffusion-weighted imaging. To the best of our knowledge, most deep-learning studies for predicting pCR have utilized only DCE-MRI, with only a few studies utilizing multiparametric MRI. For example, Dammu et al. used DCE-MRI and T2-weighted imaging, while Zhou et al. used DCE-MRI and DWI^22,24^. Third, we used post-treatment MRI data alone, without pretreatment or interim MRI data. The combined use of pre-treatment and post-treatment MRI could improve model performance, as pre-treatment MRI provides information on the location and enhancing/shape characteristics of the initial tumor. The development of deep-learning models using multiple time-point images remains understudied because of the challenges of integrating multiple channels and neural networks while maintaining consistent correlations between multiple time-point images for the same patient^14^. Previously, two studies reported that combining pre- and post-treatment MRI data improved the predictive performance for pCR compared with using single time-point data^23,24^. Owing to the lack of relevant studies, further research is needed to elucidate whether using multiparametric MRI and multiple treatment time-point images can improve the predictive performance for pCR. Fourth, the utilization of subtraction images from all dynamic phases, rather than solely from the first, third, and fifth phases as conducted in this study, may enhance prediction performance. However, due to the routine post-processing of only these phases to obtain subtraction images in our institution, they were exclusively utilized in our deep learning model. Future studies should address this consideration to potentially improve model performance. Fifth, limitations related to the segmentation method include the fact that a radiologist conducted the segmentation, raising concerns about potential intra-and inter-observer variability. Additionally, only three points (initial, middle, and end) of each lesion were marked, instead of all points, which could lead to potential erroneous segmentation. Machine-based automatic segmentation may ensure more accurate and objective results compared to the current manual method. Sixth, we did not combine MRI features with original clinical information during modeling, concerned that this approach might limit model scalability and compromise effective feature selection. However, this method could simplify the model, reduce computational burden, and potentially improve performance. Future research should explore this method. Seventh, we did not perform an independent test before building a combined model. Conducting an independent test may improve the model performance. Future studies should consider incorporating an independence test to better assess the complementarity of information and potentially enhance model robustness. Lastly, it should be mentioned that the AUC value of the single delayed-phase model, which had the highest AUC value among the models, was only 0.74. Further model improvement is necessary for clinical application. Future studies should consider training with larger sample sizes, utilizing multiparametric MRI data, combining pre-treatment and post-treatment MRI data, and employing more advanced segmentation methods.

## Conclusion

We found that the deep-learning model utilizing a single delayed-phase MRI exhibited superior performance compared to both the single early-phase MRI and the combined model incorporating multiple dynamic phases and clinical data in discriminating pCR. The application of our deep-learning model holds promise for evaluating potential candidates for obviating the need for breast surgery. However, further model improvements and validations are required to confirm their utility.

## Electronic supplementary material

Below is the link to the electronic supplementary material.

Supplementary Material 1

## Data Availability

The datasets generated and analyzed during the current study are available from the corresponding author on reasonable request.
