# Supplementary material for: Deep-learning based discrimination of pathologic complete response using MRI in HER2-positive and triple-negative breast cancer
Source: Sci Rep. 2024 Oct 4;14:23065. doi: 10.1038/s41598-024-74276-w (PMC11452398; doi:10.1038/s41598-024-74276-w)

**Supplementary Material**

**Deep-learning based discrimination of pathologic complete response using MRI in HER2-positive and Triple-negative breast cancer**

Soo-Yeon Kim, M.D. Ph.D.^1^, Jinsu Lee, M.S.^2^, Nariya Cho, M.D. Ph.D.^3,4,5^

Young-Gon Kim, Ph.D.^2,6,7^

^1^ Department of Radiology, Korea University Guro Hospital, Korea University College of Medicine, Seoul, Korea

^2^ Innovative Medical Technology Research Institute, Seoul National University Hospital, Seoul, Republic of Korea

^3^Department of Radiology, Seoul National University Hospital, Seoul, Republic of Korea

^4^Department of Radiology, Seoul National College of Medicine, Seoul, Republic of Korea

^5^Institute of Radiation Medicine, Seoul National University Medical Research Center, Seoul, Republic of Korea

^6^Department of Transdisciplinary Medicine, Seoul National University Hospital, Seoul, Republic of Korea

^7^Department of Medicine, Seoul National University College of Medicine, Seoul, Republic of Korea

of Medicine, Seoul National University College of Medicine, Seoul, Republic of Korea

Soo-Yeon Kim and Jinsu Lee contributed equally as first authors.

Corresponding author: Young-Gon Kim, Ph.D.

**Supplementary Tables**

Table E1. Dynamic contrast-enhanced MRI protocol

| Parameters | Philips Ingenia  N=471 (55%) | Siemens Skyra  N=381 (45%) |
| --- | --- | --- |
| Sequence | 3D fat-suppressed T1-weighted gradient echo sequence (eTHRIVE) | 3D fat-suppressed T1-weighted gradient echo sequence (VIBE) |
| Orientation | Axial | Axial |
| TR, msec | 4.5 | 4.7 |
| TE, msec | 2.0 | 1.7 |
| Field of view, mm^2^ | 300 × 300 | 320 × 320 |
| Matrix size | 332 × 332 | 384 × 384 |
| In-plane resolution, mm^2^ | 0.9 × 0.9 | 0.8 × 0.8 |
| Slice thickness, mm | 1.0 | 1.0 |
| Number of slices | 160 | 144 |
| Gap | 0 | 0 |
| Flip angle, degree | 12 | 10 |
| Fat suppression | SPAIR | SPAIR |
| Number of dynamics | 6 (1 pre+ 5 post-contrast) | 6 (1 pre+ 5 post-contrast) |

Note.— eTHRIVE = enhanced-T1 high-resolution isotropic volume examination, SPAIR = spectral attenuated inversion recovery, TE = echo time, TR= repetition time, VIBE = volume-interpolated breath-hold examination.

Table E2. Performance of the clinical models developed using various deep-learning and machine learning methods.

| Methods | AUC | Accuracy | Sensitivity | Specificity | PPV | NPV |
| --- | --- | --- | --- | --- | --- | --- |
| Logistic Regression | 0.59 (0.53-0.66) | 0.70 (0.64-0.75) | 0.97 (0.94-1.01) | 0.21 (0.10-0.33) | 0.68 (0.65-0.72) | 0.83 (0.62-1.04) |
| Random Forest | 0.6 (0.57-0.62) | 0.70 (0.68-0.72) | 0.98 (0.97-0.99) | 0.22 (0.16-0.28) | 0.69 (0.67-0.70) | 0.85 (0.82-0.89) |
| Support Vector Machine | 0.62 (0.57-0.66) | 0.62 (0.59-0.65) | 0.97 (0.90-1.03) | 0.02 (-0.02-0.05) | 0.63 (0.62-0.64) | 0.04 (0.04-0.13) |
| XGBoost | 0.63 (0.59-0.67) | 0.64 (0.62-0.66) | 0.89 (0.87-0.91) | 0.20 (0.15-0.25) | 0.66 (0.65-0.67) | 0.50 (0.47-0.53) |
| Multilayer Perception | 0.62 (0.57-0.68) | 0.63 (0.61-0.65) | 0.78 (0.73-0.83) | 0.36 (0.29-0.44) | 0.68 (0.66-0.70) | 0.49 (0.45-0.53) |

Note. – 95% confidence intervals in parentheses. AUC = area under the receiver operating characteristic curve, NPV= negative predictive value, PPV= positive predictive value, Ref= reference standard.

Table E3. Comparison of the two prior studies and our study for study design

|  | Qu et al.^1^ | Dammu et al.^2^ | Our study |
| --- | --- | --- | --- |
| Ground truth | Surgical pathologic results | Surgical pathologic results | Surgical pathologic results |
| Composition of breast cancer subtype | All subtypes | All subtypes | HER2 and triple-negative |
| Number of training set | 244 (non-pCR=137, pCR=107) | 152 (non-pCR=110, pCR=42) | 724 (non-pCR=464, pCR=260) |
| Number of validation set | 58 (non-pCR=33, pCR=25) | Five-fold cross-validation | 128 (non-pCR=78, pCR=50) |
| Input sequence | DCE-MRI (six phases) | DCE-MRI  (three phases) | 1^st^, 3^rd^, and 5^th^ dynamic phases of DCE-MRI (subtraction images) |
| Field strength | 1.5-T | 1.5-T | 3.0-T |
| Deep-learning models | (a) Pre-NAC model  (b) Post-NAC model  (c) Combined pre- and post-NAC model | (a) Each TP: TP1, TP2, TP3, TP4  (b) Combined TP:  TP1+TP2, TP1+TP3, TP1+TP4 | (a) Single phase  (b) Multiple phase  (c) Combined: multiple phase + clinical data |
| ROI segmentation | Performed by radiologists | Whole images without segmentation | (a) Cropped model: Performed by radiologists  (b) Uncropped model: Whole images without segmentation |
| Clinical data used | ER, PR, HER2 | Age, race, ER, PR, HER2-, Ki-67 | Age, tumor size at baseline MRI, clinical T stage, clinical N stage, ER, PR, HER2, Ki-67,histologic grade, histologic type |
| The highest AUC | 0.970 (Combined model) | 0.81 (TP1+TP4) | 0.74 (Single delayed-phase MRI model) |

Note. – AUC = area under the receiver operating characteristic curve, DCE-MRI = dynamic contrast-enhanced magnetic resonance imaging, ER = estrogen receptor, HER2 = human epidermal growth factor receptor type 2, NAC = neoadjuvant chemotherapy, PR = progesterone receptor, ROI = region-of-interest, TP = time point.

References

1 Qu, Y. H. et al. Prediction of pathological complete response to neoadjuvant chemotherapy in breast cancer using a deep learning (DL) method. Thorac Cancer 11, 651-658, doi:10.1111/1759-7714.13309 (2020).

2 Dammu, H., Ren, T. & Duong, T. Q. Deep learning prediction of pathological complete response, residual cancer burden, and progression-free survival in breast cancer patients. PLoS One 18, e0280148, doi:10.1371/journal.pone.0280148 (2023)

**Supplementary Figure**

Figure E1. Visualizing 3D convolutional neural network (CNN) layers using Grad-CAM. Results for four cases are shown, with the left column showing MRI images and the right column showing the corresponding Grad-CAM heat maps. All presented cases are true negative cases where the patient's surgical specimen (ground truth) had residual cancer and the CNN model correctly identified the residual cancer. The Grad-CAM heat map shows that the activation of the CNN model occurs in regions corresponding to residual enhancing lesions in the MRI images.


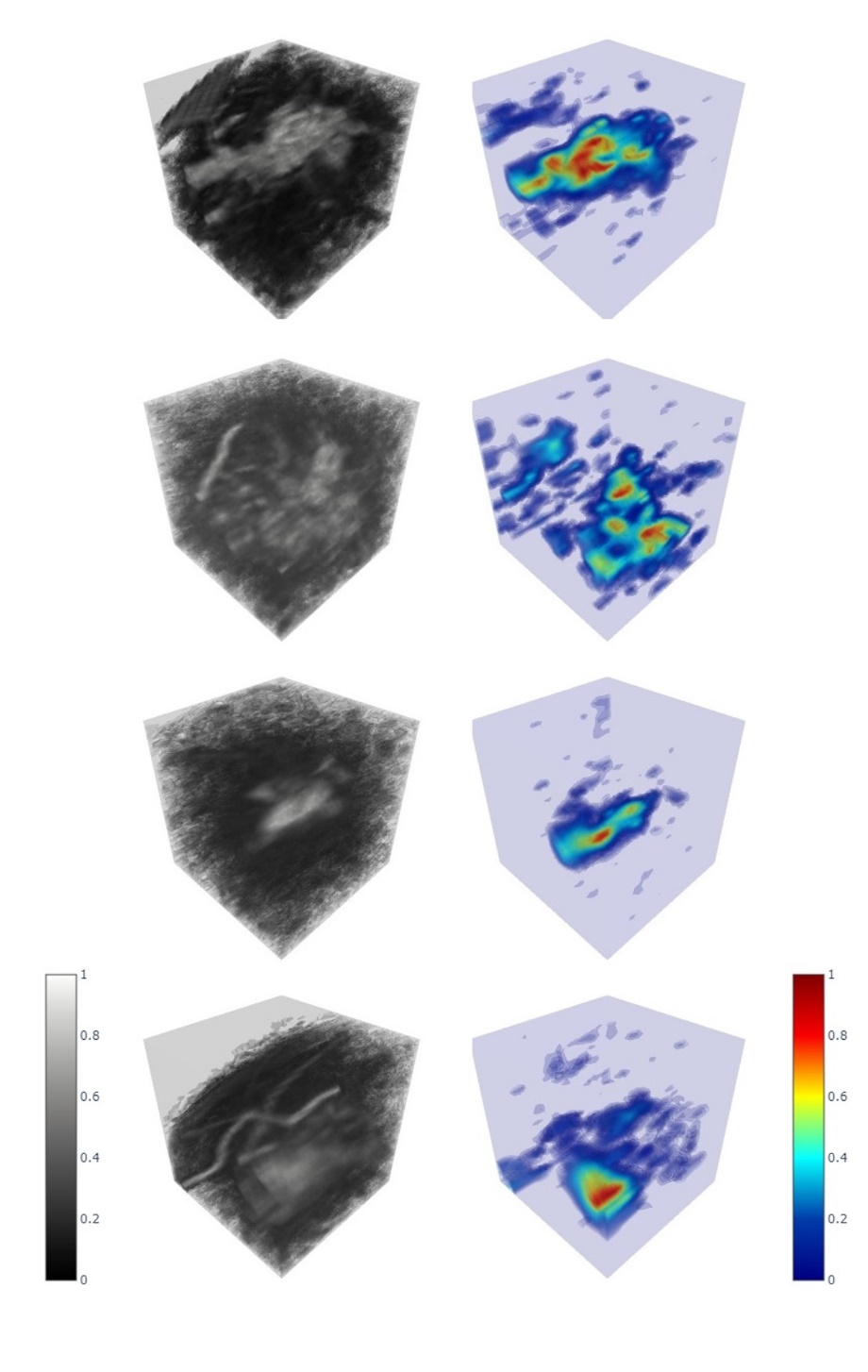

Supplement: Supplementary file 1 — Supplementary Material 1 [file 41598_2024_74276_MOESM1_ESM.docx]
